# Hypoxia aggravates ferroptosis in RPE cells by promoting the Fenton reaction

**DOI:** 10.1038/s41419-022-05121-z

**Published:** 2022-07-29

**Authors:** Yoshiyuki Henning, Ursula Sarah Blind, Safa Larafa, Johann Matschke, Joachim Fandrey

**Affiliations:** 1 Institute of Physiology, University Hospital Essen, University of Duisburg-Essen, Essen, Germany; 2 Institute of Cell Biology (Cancer Research), University Hospital Essen, University of Duisburg-Essen, Essen, Germany

**Keywords:** Cell death, Cell death in the nervous system, Visual system

## Abstract

Oxidative stress and hypoxia in the retinal pigment epithelium (RPE) have long been considered major risk factors in the pathophysiology of age-related macular degeneration (AMD), but systematic investigation of the interplay between these two risk factors was lacking. For this purpose, we treated a human RPE cell line (ARPE-19) with sodium iodate (SI), an oxidative stress agent, together with dimethyloxalylglycine (DMOG) which leads to stabilization of hypoxia-inducible factors (HIFs), key regulators of cellular adaptation to hypoxic conditions. We found that HIF stabilization aggravated oxidative stress-induced cell death by SI and iron-dependent ferroptosis was identified as the main cell death mechanism. Ferroptotic cell death depends on the Fenton reaction where H_2_O_2_ and iron react to generate hydroxyl radicals which trigger lipid peroxidation. Our findings clearly provide evidence for superoxide dismutase (SOD) driven H_2_O_2_ production fostering the Fenton reaction as indicated by triggered SOD activity upon DMOG + SI treatment as well as by reduced cell death levels upon SOD2 knockdown. In addition, iron transporters involved in non-transferrin-bound Fe^2+^ import as well as intracellular iron levels were also upregulated. Consequently, chelation of Fe^2+^ by 2’2-Bipyridyl completely rescued cells. Taken together, we show for the first time that HIF stabilization under oxidative stress conditions aggravates ferroptotic cell death in RPE cells. Thus, our study provides a novel link between hypoxia, oxidative stress and iron metabolism in AMD pathophysiology. Since iron accumulation and altered iron metabolism are characteristic features of AMD retinas and RPE cells, our cell culture model is suitable for high-throughput screening of new treatment approaches against AMD.

## Introduction

Age-related macular degeneration (AMD) is the most common blinding disease in elderly people in developed countries and represents the third leading cause for blinding worldwide [1, 2]. AMD patients develop distorted central vision which can progress to loss of central, high acuity vision caused by a gradual degeneration of photoreceptors in the macula. AMD is a complex disease with not yet fully understood pathophysiology comprising several risk factors from which oxidative stress and decreased oxygen supply, i.e. hypoxia, are considered most severe [3–5]. In the aging retina, protective mechanisms such as superoxide dismutases (SODs), glutathione peroxidases (GPXs) or catalase become less effective resulting in accumulation of oxidative damage [5–7]. Hypoxia is caused by age-related structural changes of the blood retinal barrier, leading to deteriorated supply of photoreceptors with nutrients and oxygen through retinal pigment epithelium (RPE) cells, a monolayer of pigmented cells located between the photoreceptors and the choriocapillaris. Most AMD patients (~90%) develop dry AMD or geographic atrophy which is associated with dysfunction and gradual degeneration of RPE cells, choriocapillaris, and photoreceptors. The other late form is wet or neovascular AMD, which is associated with choroidal neovascularization, vessel leakage and scarring of the retina [1, 5, 8]. Unfortunately, there is still no convincing treatment for dry AMD [9]. To develop new treatment strategies for dry AMD, it is necessary to gain deeper insights into the pathophysiological pathways leading to RPE degeneration on the molecular level. Reducing RPE damage before the transition of early AMD to late AMD could be key to prevent dry AMD, because photoreceptor degeneration in dry AMD is caused by dysfunctional RPE cells that get atrophic [7, 9, 10]. Apoptosis, necroptosis, and ferroptosis are considered to be involved in RPE atrophy and AMD pathophysiology [11–13]. Apoptosis is the classic mode of regulated cell death involving several modulators and pathways in which caspases play a crucial role. Necroptosis is regulated by successive activation of receptor interacting protein kinase 1 (RIPK1), RIPK3, and mixed lineage kinase domain like pseudokinase (MLKL) resulting in cell permeabilization and cell membrane rupture. Ferroptosis is a cell death mode associated with lipid peroxidation of polyunsaturated fatty acids leading to plasma membrane rupture. Lipid peroxidation is caused by hydroxyl radicals which are produced in the Fenton reaction where intracellular labile iron and H_2_O_2_ serve as substrates [11, 14, 15].

Many studies have already investigated modes of RPE cell death by inducing oxidative stress to RPE cells [16–20]. However, the histopathology of dry AMD suggests that oxidative stress is accompanied by hypoxia [7]. Hypoxia triggers the stabilization of hypoxia-inducible factors (HIFs), cellular oxygen sensors facilitating the adaptation to hypoxic conditions by regulating a majority of oxygen-dependent genes. HIFs are dimeric transcription factors with an oxygen-labile α-subunit and a constitutively expressed β-subunit located in the nucleus [21, 22]. Under normoxic conditions, prolyl hydroxylases named PHD1, PHD2, and PHD3 hydroxylate the α-subunit by which they are marked for proteasomal degradation. PHD activity is strictly dependent on oxygen, thus under hypoxic conditions hydroxylation stops, HIF-α subunits are stabilized, translocate to the nucleus, dimerize with the β-subunit and initiate expression of hypoxia-responsive genes [23]. To date three HIF isoforms are known from which HIF-1 and HIF-2 are considered most relevant for the hypoxia response [24]. HIFs are protective factors in the short-term but chronic hypoxia like it is observed in AMD can be detrimental due to HIF-dependent inflammation, neovascularization, metabolic shift or impaired lipid transport [3, 25–27]. Consequently, an in vitro RPE model combining oxidative stress and hypoxia would resemble the situation observed in dry AMD. To our knowledge, no such model is available thus far, limiting the knowledge on molecular pathways involved in dry AMD pathophysiology. In the present study, we used a human RPE cell line, ARPE-19, and induced oxidative stress as well as HIF stabilization in order to investigate the interplay of these two risk factors in RPE degeneration.

## Material and methods

### Reagents and Antibodies

The following non-standard reagents were used in the study: Sodium iodate (SI; sc-251029, Santa Cruz Biotechnology, Heidelberg, Germany), dimethyloxalylglycine (DMOG; Cay-71210, Cayman Chemical, Ann Arbor, MI), diethyldithiocarbamate (228680, Sigma Aldrich, Taufkirchen, Germany), phenylmethylsulfonyl fluoride (10837091001, Sigma Aldrich), Z-VAD-FMK (ab120487, Abcam, Cambridge, UK), Nec-1s (ab221984, Abcam), GSK872 (ab254395, Abcam), Necrosulfonamide (NSA) (480073, Sigma Aldrich), Ferrostatin-1 (Fer-1; ab146169, Abcam), Liproxstatin-1 (Lip-1; Cay-17730, Cayman Chemical), Deferoxamine Mesylate (ab120727, Abcam), 2’2-Bipyridyl (D216305-2, Sigma Aldrich), and DharmaFECT 4 Transfection Reagent (T-2004-02, Horizon Discovery, Waterbeach, UK). The following antibodies were used: Anti-HIF-1α (610958, BD Biosciences, Franklin Lakes, NJ), anti-HIF-2α (NB100-122, Novus Biologicals, Littleton, CO), anti-SOD2 (13141 S, Cell Signaling Technology, Danvers, MA, USA), anti-GPX1 (3206 S, Cell Signaling Technology), anti-ZIP8 (20459-1-AP, Proteintech Germany GmbH, Planegg-Martinsried, Germany), anti-DMT1 (20507-1-AP, Proteintech), anti-TFR1 (66180-1-IG, Proteintech), anti-GPX4 (ab125066, Abcam), anti-Tubulin (sc-8035, Santa Cruz), anti-ZIP14 (PA5-21077, Thermo Fisher Scientific, Waltham, MA), anti-FTH (sc-376594, Santa Cruz Biotechnology), anti-FTL (68068-1-Ig, Proteintech), anti-Actin (A2103, Sigma Aldrich), as well as goat anti-mouse (A2304, Sigma Aldrich), and goat anti-rabbit (A0545, Sigma Aldrich) secondary antibodies.

### Cell culture

Human RPE cells (ARPE-19, CRL-2302, American Type Culture Collection, Manassas, VA; distributed by LGC Standards GmbH, Wesel, Germany) were routinely cultured in a mixture of Dulbecco’s modified Eagle’s medium (DMEM)/F-12 with GlutaMAX (31331028, Thermo Fisher Scientific), supplemented with 10% fetal bovine serum and penicillin–streptomycin (full growth medium) at 37 °C under 21% O_2_ and 5% CO_2_. All experiments were conducted with DMEM/F12 supplemented with 1% FBS under 3% O_2_ (23 mmHg) in a hypoxia chamber to simulate physioxic conditions [28–30]. In order to resemble AMD pathophysiology in ARPE-19 cells, we induced oxidative stress by SI, an established oxidizing agent used to induce AMD-like phenotype in RPE cells [16, 18, 31, 32]. We further induced HIF-α stabilization by DMOG, a PHD inhibitor, which results in HIF stabilization. SI and DMOG stock solutions were prepared in DMEM/F12. Cells were authenticated by STR profiling and routinely tested for mycoplasma contamination. All experiments were conducted within five passages.

### LDH assay

To assess cell viability, LDH release was measured using a CyQUANT™ LDH Cytotoxicity Assay (C20301, Thermo Fisher Scientific). Briefly, cells were seeded at a density of 10,000 cells/well in a 96-wellplate. Confluent cells were treated for 24 h under 3% O_2_. Subsequently, 50 µL of cell culture medium was transferred to a 96-wellplate and subjected to LDH measurement according to manufacturer’s instructions. Absorbance was measured at 490 nm with a plate reader.

### Annexin V apoptosis detection assay

A PE Annexin V Apoptosis Detection Kit (559763, BD Biosciences) was used to determine the number of dead cells upon treatment with DMOG and/or SI. ARPE-19 cells were seeded in 6-wellplates at a density of 200,000 cells/well. Confluent cells were treated with SI and/or DMOG for 20 h hours. After treatment, medium was collected and centrifuged at 500 rcf for 5 min to collect already detached cells. Adherent cells were collected by trypsinization and centrifuged at 500 rcf for 5 min. Cells were subsequently washed with PBS twice and resuspended in 100 µL Annexin V binding buffer. The cell suspension was incubated for 15 min with 5 µL Annexin V conjugated to phycoerythrin (PE) and 5 µL 7-Aminoactinomycin (7-AAD). All samples were analyzed with a FACSCelesta ^TM^ Flow Cytometer (BD Biosciences).

### siRNA transfection

For siRNA knockdown of *SOD2*, ARPE-19 cells were transfected with 50 nM ON-TARGETplus Human SOD2 siRNA (L-009784-00-0005, Horizon Discovery) or scrambled control siRNA (D-001810-10-05, Horizon Discovery). In brief, at approximately 70% confluency, cells were transfected with siRNA in a mixture of DharmaFECT 4 (final dilution in well: 1:400) and OptiMEM (31985062, Thermo Fisher Scientific) diluted in DMEM/F12 supplemented with 10% FBS without the addition of antibiotics. After 6 h incubation, medium was changed to full growth medium and cells were cultured for another 48 hours before conducting experiments.

### Western blot

Cells were washed with cold PBS and collected in lysis buffer in a hypoxia chamber, incubated on ice for 20 min and centrifuged for 5 min at 500 rpm. The supernatant was collected and stored at −80 °C until use. 30 µg of total protein were incubated in Laemmli sample buffer for 5 min at 95 °C and subjected to sodium dodecyl sulfate polyacrylamide gel electrophoresis (SDS-PAGE). Separated proteins were transferred to a PVDF membrane using a Trans-Blot Turbo Transfer System (Bio-Rad Laboratories, Feldkirchen, Germany). Membranes were blocked with 5% non-fat dry milk in TBS-T for 1 h at room temperature. Primary antibodies were diluted in blocking buffer and incubated overnight at 4 °C. Secondary antibodies were diluted in blocking buffer (1:10,000) and incubated 1 h at room temperature. Signals were developed with SuperSignal West Femto Maximum Sensitivity Substrate (34096, Thermo Fisher Scientific) and detected with a Fusion FX System (Vilber, Eberhardzell, Germany).

### Quantitative Real-Time PCR

Total RNA was isolated using the NucleoSpin RNA kit (740955.250, MACHEREY-NAGEL, Düren Germany) according to the manufacturer’s instructions. Complementary DNA (cDNA) was synthesized from 500 ng total RNA using M-MLV reverse transcriptase (M1705, Promega, Walldorf, Germany) and oligo dT primer. Quantitative Real-Time PCR (qRT-PCR) was performed with a Biozym Blue S’Green master mix (331416XL, Biozym Scientific, Hessisch Oldendorf, Germany) on a BioRad IQ5 Real-Time PCR Detection System. Relative expression levels were calculated with the ∆∆ct method [33] using *hypoxanthine-guanine phosphoribosyltransferase* (*HPRT)* as reference gene. The following primer pairs were used: *GPX1* 5′-AGTCGGTGTATGCCTTCTCG-3′ forward and 5′- TCTTGGCGTTCTCCTGATGC-3′ reverse; *GPX4* 5′-GTGGAAGTGGATGAAGAT-3′ forward and 5′-GATGAGGAACTTGGTGAA-3′ reverse; *SOD1* 5′-AGGCATGTTGGAGACTTGGG-3′ forward and 5′-TGCTTTTTCATGGACCACCAG-3′ reverse; *SOD2* 5′-CGTTGGCCAAGGGAGATGTT-3′ forward and 5′-CACGTTTGATGGCTTCCAGC-3′ reverse; *DMT1* 5′-CTTTGCCAATGGACTAGGCT-3′ forward and 5′-CTTCTGTCAGCAGGCCTTTAG-3′ reverse; *TFR1* 5′-ACTTCTTCCGTGCTACTTCCAG-3′ forward and 5′-ACTCCACTCTCATGACACGATC-3′ reverse; *ZIP8* 5′-ATGCTACCCAAATAACCAGC-3′ forward and 5′-CAGGAATCCATATCCCCAAAC-3′ reverse; *ZIP14* 5′-TAAGCAGAAAAATGAGCATC-3′ forward and 5′-ACCTTTCAGCCAGTAGCAAG-3′ reverse; *HPRT* 5′-CCTGGCGTCGTGATTAGTGA-3′ forward and 5′-CGAGCAAGACGTTCAGTCCT-3′ reverse.

### Superoxide dismutase activity assay

Superoxide dismutase (SOD) activity in ARPE-19 cells was measured using a colorimetric assay (19160, Sigma Aldrich). Measurements were conducted according to manufacturer’s instructions. Briefly, cells were seeded in 6-wellplates at a density of 200,000 cells per well. Upon confluence, cells were treated with SI and/or DMOG for 16 h at 3% O_2_. After treatment, cells were lysed using an ice-cold lysis buffer containing 0.1 mM Tris/HCl (pH 7.4), 0.5% Triton X-100, 5 mM β-Mercaptoethanol, and 0.1 mg/mL phenylmethylsulfonyl fluoride and centrifuged to collect the supernatant. One part of each sample was measured directly, and the other part was incubated with 2 mM diethyldithiocarbamate to chelate Cu to inactivate SOD1 in order to measure SOD2 activity. All samples were measured in triplicate and absorbance was read at 450 nm. SOD activities were normalized to total protein content.

### Image-iT lipid peroxidation assay

Cells were seeded at 40,000 cells/well in 24-wellplates and treated with SI and/or DMOG the next day for 16 h under 3% O_2_ in a hypoxia chamber. After treatment, cells were washed with warm culture medium three times and incubated for 30 min under 3% O_2_ with 10 µM Image-iT lipid peroxidation sensor (C10445, Thermo Fisher Scientific). After incubation, cells were washed with PBS and fixed with 4% paraformaldehyde for 15 min at 3% O_2_. The Image-iT lipid peroxidation sensor is based on BODIPY 581/591 C11 which has a fluorescence emission peak at approximately 590 nm in its native form. Upon oxidation, the emission shifts to approximately 510 nm. Fluorescence signal was detected using an Eclipse Ts2-FL fluorescence microscope (Nikon, Amsterdam, Netherlands) and quantified with IMAGEJ.

### Detection of Fe^2+^ and Fe^3+^ using flow cytometry

Cells were plated in 6-wellplates at a density of 200,000 cells/well. Confluent cells were treated with SI and/or DMOG and kept in hypoxic conditions (3% O_2_) for 8 h or 16 h. For Fe^2+^ quantification, cell pellets were stained with 10 µM Rhodamine B-[(1,10-phenanthroline-5-yl)-aminocarbonyl]benzylester (RPA, Squarix Biotechnology, Marl, Germany) for 12 min, washed with PBS and incubated in PBS for 15 min at 37 °C. For Fe^3+^ quantification, cell pellets were stained with 10 µM 7-Nitrobenz-2-oxa-1,3-diazole-desferrioxamine (NDB-DFO, Squarix Biotechnology, Marl, Germany) for 12 min, washed with PBS and incubated in PBS for 15 min at 37 °C. The mean fluorescence intensity (MFI) was detected by flow cytometry (BD CytoFLEX S, Beckman Coulter; FL-2). Fold changes were quantified to the corresponding non-treated controls as previously described [34]. In brief, RPA fluorescence is quenched by Fe^2+^ allowing the determination of iron reduction [35, 36], and NBD-DFO fluorescence is quenched by Fe^3+^ allowing the determination of ferric iron [37].

### Statistical analyses

Statistical analyses were performed using GraphPad Prism software (Version 8.0, San Diego, CA, USA). Analyses were conducted using two-tailed unpaired *t*-test, one-way ANOVA with Tukey’s multiple comparisons test or two-way ANOVA with Tukey’s multiple comparisons test according to the recommendations of Lord et al. [38]. Western blot data were normalized to each control group to calculate the fold-change and log-transformed prior to analyses. All data are expressed as mean+SD. Sample sizes were chosen according to the recommendations of Naegle et al. [39] and are indicated in the respective figure legends. Statistical significance was defined as ns = *p* > 0.05; *p < 0.05; **p < 0.01, ***p < 0.001, and ****p < 0.0001.

## Results

### HIF stabilization aggravates oxidative damage

**Fig. 1:**
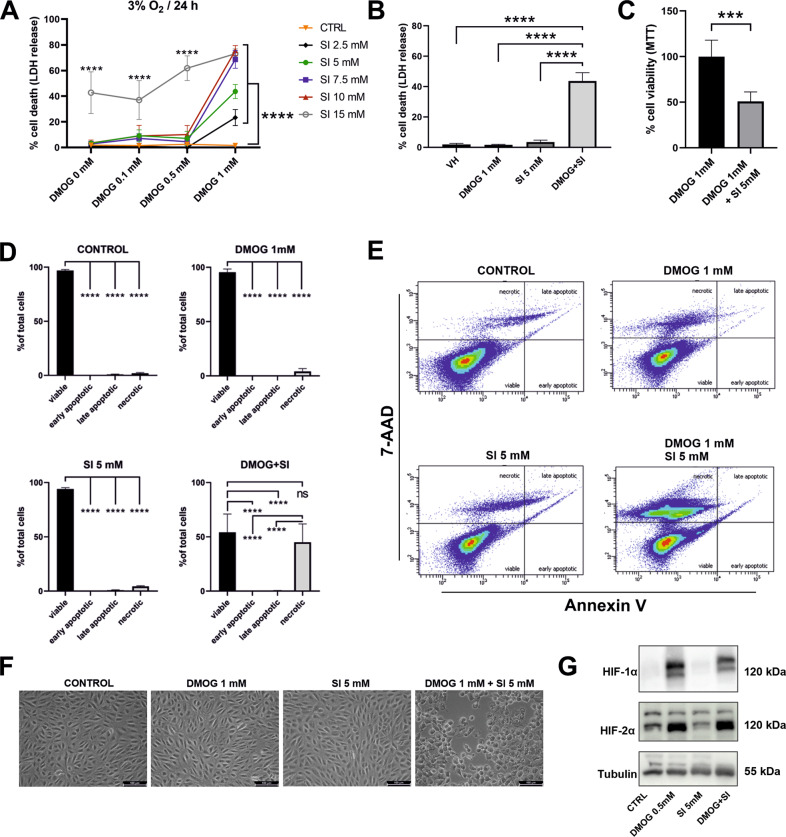
HIF stabilization aggravated SI-induced cell death in ARPE-19. **A** ARPE-19 cells were treated with different concentrations of SI, DMOG or a combination of both compounds for 24 h under 3% O_2_ and cell death was assessed by measuring LDH release (*N* = 5-6). **B** A subset of LDH data showing cell death in ARPE-19 cells treated with 1 mM DMOG, 5 mM SI, and co-treatment with DMOG and SI where approximately 50% of cells were dead after 24 h (**B**). Cell death rate was further confirmed by **C** MTT assay and **D** FACS Annexin V assay. **E** Histograms of FACS Annexin V-PE and 7-AAD stained cells treated with DMOG and/or SI. **F** Microscopic evaluation confirmed advanced cell death by co-treatment with 1 mM DMOG and 5 mM SI. **G** HIF-1α and -2α protein levels were increased by DMOG treatment as well as co-treatment with SI for 24 h under 3% O_2_. Data are presented as means + SD. LDH, MTT, and FACS assays (5–6 independent groups) were statistically analyzed with two-way ANOVA, *t*-test, and one-way ANOVA, respectively. ns = not significant, ***p < 0.001, and ****p < 0.0001. Scale bar = 100 μm.

We treated ARPE-19 cells with 0, 2.5, 5, 7.5, 10, 15 mM SI with or without DMOG in a hypoxia chamber set to 3% O_2_ and LDH release was determined as a measure of cell death. Cells treated with SI alone required 15 mM SI to detect significant cell death after 24 h (Fig. 1A). However, when HIFs were additionally stabilized by 1 mM DMOG, cell death was already significantly upregulated by 2.5 mM SI after 24 h while treatment with 1 mM DMOG alone had no effect on cell viability (Fig. 1A). In all SI-treated groups, co-treatment with 1 mM DMOG exacerbated cell death rate in a dose-dependent manner compared to the corresponding SI-only group. We further validated the cell death rate measured by LDH assay by two independent methods using 5 mM SI and/or 1 mM DMOG which represented concentrations that induced cell death in 43.7 ± 5.5% of cells measured by LDH release (Fig. 1B). Cell viability determined by MTT assay was decreased by 50.9 ± 10.4% in the DMOG + SI-treated cells compared to DMOG-treated cells (Fig. 1C). Annexin V assay revealed that 45.1 ± 16.8% of cells showed a necrotic phenotype when cells were treated with SI + DMOG for 20 h (Fig. 1D, E). Based on these findings, we chose a concentration of 5 mM SI and 1 mM DMOG for further viability experiments, representing a treatment regimen that yielded a reproducible cell death rate determined by three quantitative methods and microscopic observation (Fig. 1F). For mRNA and protein extraction, we reduced DMOG concentration to 0.5 mM to induce HIF stabilization (Fig. 1G, Supplementary File 1) but avoid cell death.

### Ferroptosis is the main cell death pathway

**Fig. 2:**
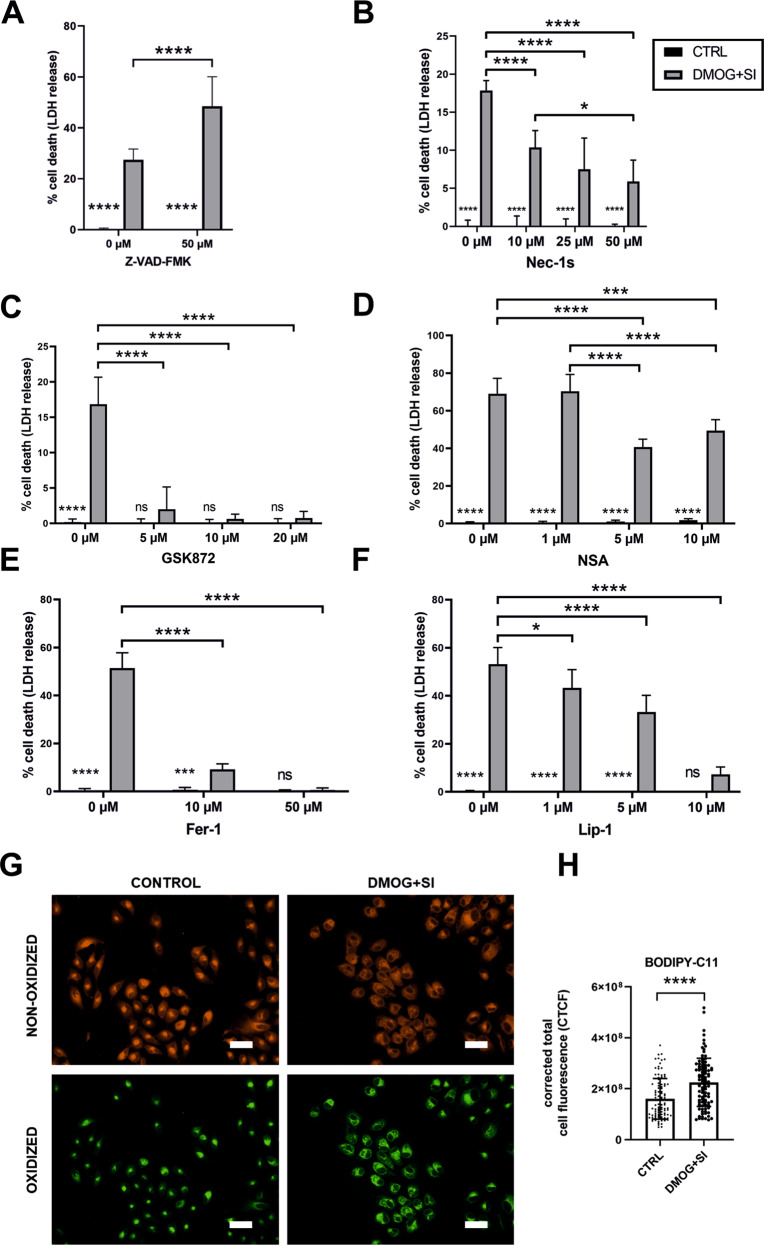
Ferroptosis inhibitors rescued cells from cell death. ARPE-19 cells were treated with DMOG and SI and co-treated with inhibitors of apoptosis, necroptosis, and ferroptosis. Treatment with **A** the apoptosis inhibitor Z-VAD increased DMOG + SI-induced cell death. **B** RIPK1 inhibitor Nec-1s, **C** RIPK3 inhibitor GSK872, and **D** MLKL inhibitor necrosulfonamide (NSA) are efficient inhibitors of necroptosis, but only RIPK3 inhibition by GSK872 fully rescued cells from cell death. Both ferroptosis inhibitors **E** Fer-1 and **F** Lip-1 fully rescued ARPE-19 cells from DMOG + SI-induced cell death. To further confirm ferroptotic cell death, cells were stained with the lipid peroxidation probe C11-BODIPY581/591 which revealed mainly perinuclear localization of lipid peroxidation-induced green fluorescence (**G**). **H** Quantification of cell fluorescence revealed significantly higher corrected total cell fluorescence in DMOG + SI treated cells compared to controls. Data are presented as means + SD. Inhibition assays were conducted with 6 independent groups (except for NSA treatment; *N* = 4) and statistical significance was calculated with two-way ANOVA. C11-BODIPY581/591 staining was assessed in 3 independent groups (*n* = 100) and statistical analysis was conducted with *t*-test. ns not significant, *p < 0.05, ***p < 0.001, and ****p < 0.0001. Scale bar = 50 μm.

In order to identify the cell death mechanism, we treated the cells with inhibitors of the apoptosis, necroptosis, and ferroptosis pathway and subjected the cells to DMOG + SI co-treatment to assess cell viability by LDH assay. Cells treated with the pan-caspase inhibitor Z-VAD-FMK, a well-established apoptosis inhibitor, displayed increased cell death when co-treated with DMOG and SI compared to controls (Fig. 2A) suggesting that apoptosis is not responsible for increased cell death; this is in line with Annexin V FACS data where almost no apoptotic cells were detected (Fig. 1D, E). The necroptosis pathway was analyzed by inhibiting RIPK1 with Nec-1s, RIPK3 with GSK872, and MLKL with NSA. Nec-1s significantly reduced cell death (Fig. 2B) and GSK872 fully rescued cells treated with DMOG + SI (Fig. 2C). However, inhibition of MLKL activation by NSA, which represents the final step in necroptosis execution, led to a statistically significant reduction of cell death, but still the majority of cells were dead, although very high concentrations of NSA were used (Fig. 2D). To test the involvement of ferroptosis, cells were treated with Fer-1 and Lip-1, which most likely inhibit ferroptosis by a radical-trapping mechanism reducing lipid peroxidation [40]. Both inhibitors were able to fully rescue cells from cell death (Fig. 2E, F), suggesting that ferroptotic cell death is the major cell death mechanism observed in our model. Ferroptosis was further confirmed with an oxidation-sensitive fluorescent lipid peroxidation probe based on C11-BODIPY581/591 which shifts its fluorescence emission from red to green upon oxidation. The green fluorescence localized mainly perinuclear which is typical for this probe in the oxidized state (Fig. 2G) and background-corrected total cell fluorescence was significantly higher in cells treated with DMOG + SI compared to controls (Fig. 2H), suggesting that cell death is induced by lipid peroxidation which is typical for ferroptosis.

### SOD activity paradoxically contributed to cell death

**Fig. 3:**
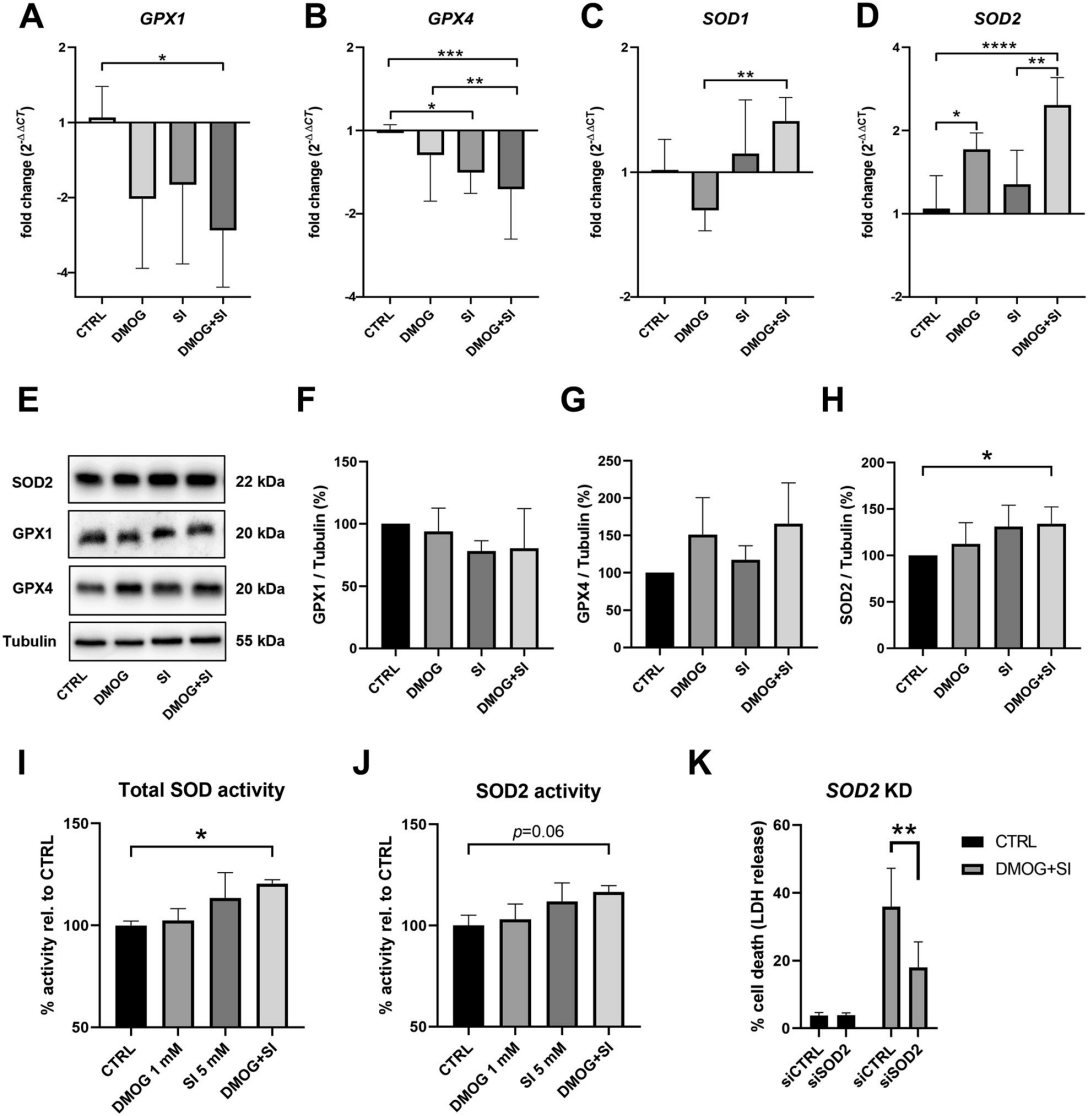
Analysis of antioxidant mechanisms revealed upregulated SOD activity, which contributed to cell death. **A**–**D** *GPX1*, *GPX4*, *SOD1*, *SOD2* transcript levels were determined by qRT-PCR in DMOG and/or SI treated ARPE-19 cells. While *GPX1* and *GPX4* expression levels were mainly decreased by the treatments, especially *SOD2* expression was upregulated by DMOG and DMOG + SI co-treatment compared to controls. **E**–**H** To validate if transcript levels reflected protein levels, GPX1, GPX4, and SOD2 protein levels were analyzed by Western blot. Only SOD2 protein levels were significantly increased by DMOG + SI co-treatment, which was also reflected by higher SOD activities (**I**, **J**). **K** In contradiction to the protective effect of SODs, *SOD2* knockdown (*SOD2* KD) rescued cells from cell death suggesting that SOD activity contributes to DMOG + SI-induced cells death. Data are presented as means + SD. Western blots (3-4 independent groups) and qRT-PCR (6 independent groups) were statistically analyzed with one-way ANOVA. *SOD2* knockdown effects (6 independent groups) were analyzed with two-way ANOVA. *p < 0.05, **p < 0.01, ***p < 0.001, and ****p < 0.0001.

GPXs and SODs are important antioxidant enzymes. SODs catalyze the dismutation of superoxide (O_2_^−^) into oxygen and hydrogen peroxide (H_2_O_2_). H_2_O_2_ is then broken down by GPX1 and catalase for complete detoxification. Moreover, GPX4 protects from lipid peroxidation, i.e. ferroptosis. Since SI treatment induces O_2_^−^ accumulation [41], GPXs and SODs are crucial in protection against SI-induced oxidative damage. Gene expression levels of *GPX1* and *GPX4* were significantly downregulated in DMOG + SI co-treated cells (Fig. 3A, B). While *SOD1* transcript levels were only higher in DMOG + SI co-treatment compared to SI treatment (Fig. 3C), *SOD2* transcription was higher in co-treated cells compared to controls and SI treatment (Fig. 3D). In contrast to transcript levels, GPX1 and GPX4 protein levels did not differ between the treatment groups (Fig. 3E–G). On the other hand, SOD2 protein levels were significantly upregulated in DMOG + SI co-treated cells compared to controls (Fig. 3E, H). To get insights if the dismutation potential follows protein levels, we measured SOD activity with a colorimetric assay. Total SOD activity was significantly upregulated in DMOG + SI co-treatment compared to controls (Fig. 3I). SOD2 activity, measured after inactivation of SOD1 by Cu-chelation, only showed a statistical trend towards higher activity in the DMOG + SI group (Fig. 3J). These results suggest that SOD activity contributes to ferroptotic cell death induced by DMOG + SI co-treatment by producing H_2_O_2_, which is a substrate of the Fenton reaction. To test this, we conducted siRNA knockdown of *SOD2* by which cell death was significantly decreased under DMOG + SI co-treatment confirming that SOD2 contributes to cell death (Fig. 3K).

### Importers for non-transferrin-bound Fe^2+^ were upregulated in DMOG + SI co-treated cells

**Fig. 4:**
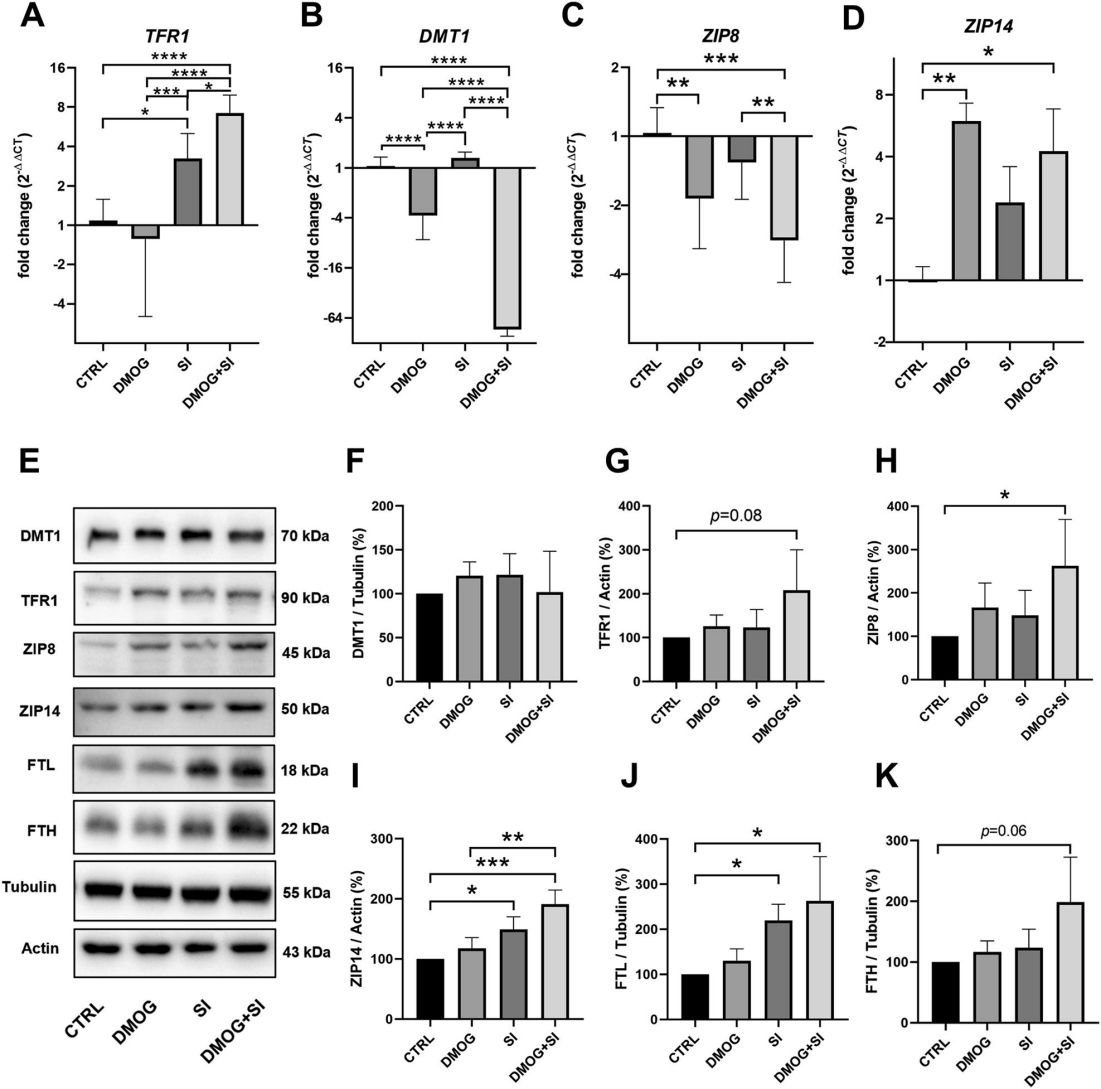
Iron transporters involved in non-transferrin-bound Fe^2+^ import were upregulated in DMOG + SI co-treated ARPE-19 cells. Transcript levels of **A** *TFR1* were significantly upregulated and **B** *DMT1* were significantly downregulated compared to all treatments. Gene expression of non-transferrin-bound iron importers **C** *ZIP8* was significantly downregulated and **D** *ZIP14* was upregulated by DMOG + SI co-treatment compared to controls. **E**–**I** Analysis of iron importers on protein level revealed that only ZIP8 and ZIP14 were significantly upregulated by DMOG + SI co-treatment. **J** The ferritin subunit FTL was significantly upregulated in SI- and DMOG + SI co-treated cells, while the other subunit FTH showed a trend towards upregulation in DMOG + SI co-treated cells (**K**). Data are presented as means + SD. Western blots (3 independent groups) and qRT-PCR (6 independent groups) were statistically analyzed with one-way ANOVA. *p < 0.05, **p < 0.01, ***p < 0.001, and ****p < 0.0001.

While gene expression of the transferrin receptor 1 (*TFR1*) was significantly upregulated by SI, DMOG, and DMOG + SI co-treatment (Fig. 4A), gene expression of the divalent metal transporter 1 (*DMT1*) was downregulated approximately 64-fold compared to controls (Fig. 4B). The two non-transferrin-bound Fe^2+^ importers *ZIP8* and *ZIP14* were expressed in a HIF-dependent but opposite manner. While DMOG and DMOG + SI co-treatment led to *ZIP8* downregulation (Fig. 4C), *ZIP14* was significantly upregulated in these groups (Fig. 4D). Protein levels revealed different patterns compared to transcript levels (Fig. 4E). TFR1 and DMT1 were not significantly changed by the treatments (Fig. 4F, G, Supplementary File 1), despite of a trend in TFR1 protein towards higher levels in co-treated cells (*p* = 0.08). In contrast, ZIP8 and ZIP14 protein levels were significantly higher when treated with DMOG + SI (Fig. 4H, I, Supplementary File 1). ZIP14 was also significantly upregulated by SI treatment. We also analyzed ferritin light chain (FTL) and ferritin heavy chain (FTH) protein, the two subunits of ferritin. FTL was significantly upregulated by SI alone and SI + DMOG co-treatment (Fig. 4J, Supplementary File 1) and FTH showed a trend towards higher levels in DMOG + SI co-treatment (Fig. 4K, Supplementary File 1).

### Fe^2+^ but not Fe^3+^ chelation fully rescued cells from ferroptotic cell death

**Fig. 5:**
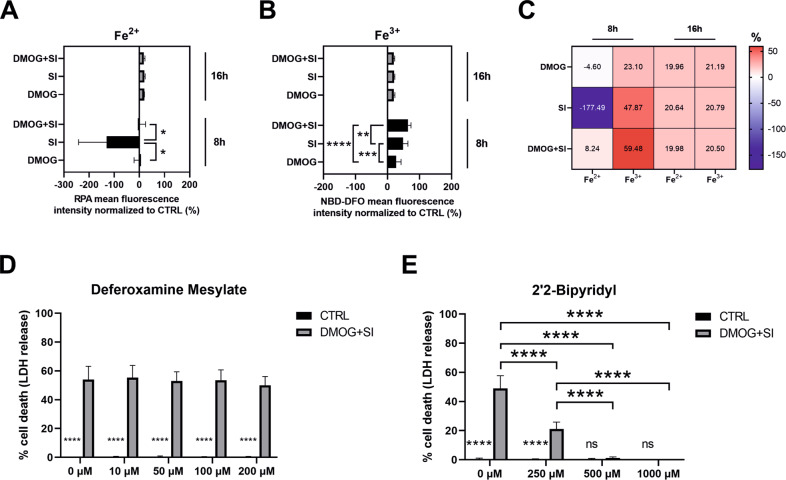
DMOG + SI co-treatment is likely to promote the Fenton reaction in DMOG + SI co-treated ARPE-19 cells. **A**–**C** The Fe^2+^-specific fluorescence sensor RPA revealed significantly lower Fe^2+^ levels in SI-treated cells compared to controls after 8 h at 3% O_2_ while no difference between controls and DMOG + SI co-treated cells were detected. In contrast, Fe^3+^ levels were significantly increased by DMOG + SI co-treatment compared to the other treatment groups after 8 h, which was assessed with the Fe^3+^-specific fluorescence sensor NBD-DFO. After 16 h, iron levels were similar in all treatment groups. While chelation of Fe^3+^ by Deferoxamine Mesylate (**D**) did not improve cell viability, Fe^2+^ chelation by 2’2-Bipyridyl **E** resulted in full rescue of cells suggesting that Fe^2+^ plays a detrimental role in DMOG + SI-induced ferroptosis. Data are presented as means + SD. RPA and NBD-DFO assays (3 independent groups) were statistically analyzed with one-way ANOVA and LDH assays were analyzed with two-way ANOVA. *SOD2* knockdown (6 independent groups) were analyzed with two-way ANOVA. ns not significant, *p < 0.05, **p < 0.01, ***p < 0.001, and ****p < 0.0001.

In order to understand how the differential iron transporter expression influences iron content of ARPE-19 cells, we measured Fe^2+^ and Fe^3+^ levels by flow cytometry. SI treatment alone for 8 h led to a significant decrease in intracellular Fe^2+^ levels. Interestingly, Fe^3+^ levels were already elevated by SI or DMOG treatment alone but displayed the highest levels in DMOG + SI co-treated cells (Fig. 5A–C). Thus, our data suggest that either Fe^3+^ import is higher or oxidation of Fe^2+^ to Fe^3+^ is increased in the SI + DMOG co-treated group due to induced Fenton reaction. To test, if Fe^3+^ or Fe^2+^ levels are involved in cell death, we treated ARPE-19 cells with an Fe^3+^ chelator Deferoxamine Mesylate and an Fe^2+^ chelator 2’2-Bipyridyl along with DMOG + SI co-treatment. While Deferoxamine Mesylate did not reduce LDH release, i.e. cell death, even at very high concentrations (Fig. 5D), 2’2-Bipyridyl reduced cell death in a concentration-dependent manner and fully rescued cells at concentrations of 500 µM and 1 mM (Fig. 5E).

## Discussion

The pathophysiology of AMD is complex and involves many risk factors. The primary factor is advanced age due to its association with decreasing antioxidant defense mechanisms and structural changes in the blood retinal barrier favoring chronic hypoxia in the macular region. To date many studies have used SI to induce ROS production in RPE cells because SI treatment leads to RPE atrophy and secondary photoreceptor degeneration resembling AMD pathophysiology in vivo. Therefore, SI treatment has been widely used as a model for dry AMD in vivo and in vitro [16, 18, 20, 41–45]. However, inducing oxidative stress alone does not fully resemble age-related morphological changes contributing to AMD pathophysiology, particularly chronic hypoxia-associated effects. Therefore, we aimed to develop an in vitro RPE model using ARPE-19 cells to investigate the role of HIFs in oxidative stress-induced RPE degeneration and the cell death pathways involved in RPE atrophy to provide new approaches for future treatment strategies for dry AMD.

To our surprise, SI-induced cell death was substantially increased when HIFs were stabilized by DMOG, suggesting that HIF stabilization aggravates oxidative stress-induced cell death. We found that Fer-1 and Lip-1, two highly potent ferroptosis inhibitors fully rescued ARPE-19 cells co-treated with DMOG and SI. This is in line with two studies in which oxidative stress induced by *tert*-butyl hydroperoxide or SI resulted in ferroptotic cell death in ARPE-19 cells [43, 46]. We also found higher levels of the oxidized form of the lipid peroxidation sensor BODIPY-C11 to be localized in the perinuclear space of DMOG + SI co-treated cells which is typically found in cells facing lipid peroxidation, the critical step of ferroptosis [47]. In line with our findings, lipid peroxidation in the context of AMD was already reported before ferroptosis was established as a cell death pathway [48]. However, we also found that the RIPK3/necroptosis inhibitor GSK872, completely inhibited cell death, while RIPK1 (by Nec-1s) and MLKL (by NSA) inhibition decreased cell death but were not able to rescue cells as it would be expected if necroptosis was the major cell death pathway. This finding is in contrast to a study where SI was reported to induce necroptosis in ARPE-19 cells and mouse RPE [16]. However, the authors used Nec-1 (not Nec-1s, which is more selective for RIPK1), a RIPK1 inhibitor with ferroptosis-associated off-target effects [49] and inhibition of MLKL, the final step of the necroptosis pathway, was not conducted. On the other hand, the authors reported an involvement of RIPK3, which was shown by e.g. formation of RIPK3 aggregates. A crosstalk between necroptosis and ferroptosis was already reported which might explain rescue of ARPE-19 cells by RIPK3 inhibition although necroptosis is not the major death pathway [50]. Consequently, our results make it most likely that HIF stabilization under oxidative stress conditions aggravates ferroptotic cell death resembling the pathophysiology of dry AMD.

It must be noted that DMOG stabilizes both, HIF-1α and HIF-2α. Previous studies have demonstrated that these two HIF isoforms play differential roles in ferroptosis with partially contradicting results. For instance, HIF-2α was associated with higher ferroptosis susceptibility in colorectal cancer cells, in murine colon tumors [51] and clear cell carcinoma cells [52] by regulation of cellular lipid and iron metabolism. However, HIF-2 signaling was also reported to reduce oxidative stress by inducing the transcription of nucleoside diphosphate-linked moiety X-type motif 1 in clear cell renal cell carcinoma, which promoted tumor growth [53]. HIF-1α, on the other hand, was attributed a protective effect against ferroptosis in several cancer cells by reducing iron and ROS levels [54], promoting lipid storage and regulating lipid metabolism [55–57]. In line with previous observations, high expression of the HIF-1 target gene carbonic anhydrase 9 had protective effects against ferroptosis in a model of malignant mesothelioma [58]. Moreover, in ferroptosis-induced brain injury and liver fibrosis models, HIF-1α suppressed ferroptosis [59, 60]. However, in non-small cell lung cancer cells and ES-2 ovarian cancer cells, HIF-1α aggravated ferroptotic cell death. Similarly, a study in a mouse model of diabetic renal tubular injury indicated that ferroptosis was aggravated by activation of the HIF-1α/ heme oxygenase-1 pathway [61]. Moreover, ZIP8 and ZIP14 were reported to be upregulated in a HIF-1α-dependent manner [62], which could have contributed to increased Fe^2+^ import in our cell model as well. Taken together, HIF signaling influences the extent of ferroptotic cell death but the differential roles of HIF-1α and HIF-2α in ferroptosis regulation turn out very differently depending on the type of disease and tissue. Thus, as a future perspective, targeted manipulation of HIF-1 and/or HIF-2 signaling in AMD-RPE models are needed to reveal novel therapeutic opportunities.

Based on our findings, we propose two crucial steps leading to ferroptosis aggravation with HIF stabilization: First, we found increased SOD activity combined with unaffected GPX1 and GPX4 levels which likely led to higher H_2_O_2_ levels because GPXs could not compensate for increased H_2_O_2_ production by SODs. In line with our findings, higher SOD activity paired with decreased GPX activity in human erythrocytes was correlated with a significantly higher risk for AMD [63]. Elevated SOD activity paired with unchanged GPX1 activity have also been reported in fibromyalgia patients, a pain syndrome associated with increased lipid peroxidation [64, 65]. Moreover, several studies have demonstrated a peroxidative rather than antioxidative role of SODs under specific circumstances: SOD2 overexpression led to an increase of H_2_O_2_ in fibrosarcoma cells [66] and SOD2 displays peroxidase activity in presence of high H_2_O_2_ concentrations [67]. Under manganese-deficient conditions, SOD2 can incorporate iron instead of manganese, resulting in an alternative SOD2 isoform with peroxidase activity [68]. In all these cases, cellular energy metabolism was impaired, similar to data obtained from RPE cells from AMD patients [69]. In addition, SOD2 exacerbated ischemia-reperfusion damage in isolated hearts when administered at high doses [70] and elevated SOD1 activity was demonstrated to be responsible for oxidative stress, protein oxidation, lipid peroxidation, presumably leading to mitochondrial dysfunction and accelerated cellular senescence in Down’s syndrome [71–73]. It must be noted that under physiological conditions, low SOD levels are sufficient to dismutate most O_2_^−^. Thus, increased SOD activity does not necessarily increase H_2_O_2_ levels [74]. However, a central pathophysiological characteristic of AMD is the accumulation of lipofuscin, a potent photoinducible ROS generator, which is associated with increased levels of O_2_^−^, singlet oxygen, H_2_O_2_ and lipid hydroperoxides [75]. In our AMD cell culture model, we used SI, which resembles lipofuscin-induced O_2_^-^ production. Consequently, elevated SOD activity along with unchanged or even decreased GPX activity could represent a central pathophysiological mechanism leading to photoreceptor damage.

Second, increased abundance of the Fe^2+^ importers ZIP8 and ZIP14 was described to lead to higher non-transferrin-bound iron import [76, 77]. While the main regulators of transferrin-bound iron import, TFR1 and DMT1 were not differently regulated on protein level, ZIP8 and ZIP14 were upregulated by DMOG + SI co-treatment, suggesting that Fe^2+^ import was increased. While the transferrin-bound iron import can be negatively regulated by intracellular iron levels [77], which was also observed on *DMT1* gene expression level in the present study, ZIP8 and ZIP14 protein levels were reported to be positively regulated by intracellular iron levels in the mouse retina [77] which corroborates our findings. Measurement of Fe^2+^ and Fe^3+^ levels revealed that Fe^2+^ levels were decreased in SI-treated cells presumably due to decreased transferrin-bound iron import. Fe^3+^ levels were increased by DMOG + SI co-treatment suggesting that Fe^2+^ to Fe^3+^ oxidation could be upregulated due to the Fenton reaction where oxidation of Fe^2+^ leads to the generation of hydroxyl radicals. Besides the above-mentioned iron transporters, a process known as ferritinophagy contributes to the intracellular iron pool as well. Ferritinophagy is an autophagy-related process which releases iron from ferritin. Thus, dysregulation of ferritinophagy can promote ferroptosis. Hypoxia and HIF-1α were reported to inhibit ferritinophagy in macrophages and osteoclasts [78, 79], while HIF-2 signaling increased expression of nuclear receptor coactivator 4, which promotes the degradation of ferritin in autophagosomes, in mouse enterocytes [80, 81]. Interestingly, we did not see differences in FTL or FTH protein levels in DMOG-treated cells, presumably because HIF-1 and -2 effects neutralized each other. In SI and DMOG + SI co-treated cells, FTL was significantly upregulated, which was most likely a result of increased intracellular iron [82]. Still, it was not sufficient to avoid ferroptotic cell death. To test whether non-transferrin-bound Fe^2+^ import and not transferrin-bound Fe^3+^ import was responsible for ferroptosis aggravation in DMOG + SI co-treated cells, we chelated Fe^2+^ with 2’2-Bipyridyl that resulted in full rescue of cells. In contrast, Fe^3+^ chelation did not influence cell death, which is in contrast to studies where the Fe^3+^ chelator Deferoxamine was used to inhibit ferroptosis [43, 83]. This suggests that the combination of oxidative stress and hypoxia induces ferroptosis by a hitherto unknown pathway, where SODs and non-transferrin-bound iron import play a pathophysiological role.

Taken together, our study provides a novel link between hypoxia, oxidative stress and iron metabolism in AMD pathophysiology. Our data demonstrate for the first time that HIF stabilization under oxidative stress conditions aggravates the Fenton reaction in RPE cells. Our findings are in line with reports of increased iron levels in the retina, RPE and Bruch’s membrane of AMD patients [84–90] and studies where iron reduction led to decreased cell death in RPE cells [91–93] implying that dry AMD pathophysiology was resembled in our cell culture model. Thus, our model provides a unique opportunity to test different compounds reducing iron overload and ferroptosis in a high throughput approach.

## Supplementary information

Supplementary File 1

aj-checklist

## Data Availability

The numerous datasets generated in the current study will be made available upon request.
