# Supplementary material for: Hypoxia aggravates ferroptosis in RPE cells by promoting the Fenton reaction
Source: Cell Death Dis. 2022 Jul 29;13(7):662. doi: 10.1038/s41419-022-05121-z (PMC9338085; doi:10.1038/s41419-022-05121-z)
Supplement: Supplementary file 1 [file 41419_2022_5121_MOESM1_ESM.pdf]

Hypoxia aggravates ferroptosis in RPE cells by promoting the Fenton reaction

Yoshiyuki Henning, Ursula Sarah Blind, Safa Larafa, Johann Matschke, Joachim Fandrey

Supplementary File 1

Full length Western Blots (Figure 1)

HIF-1α

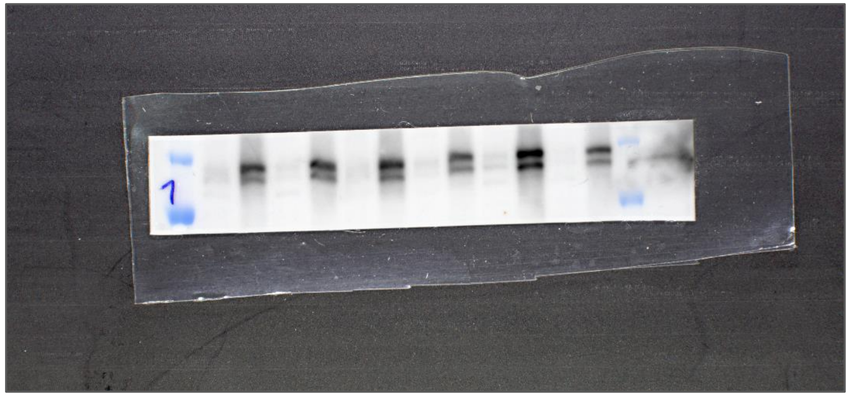

HIF-2α

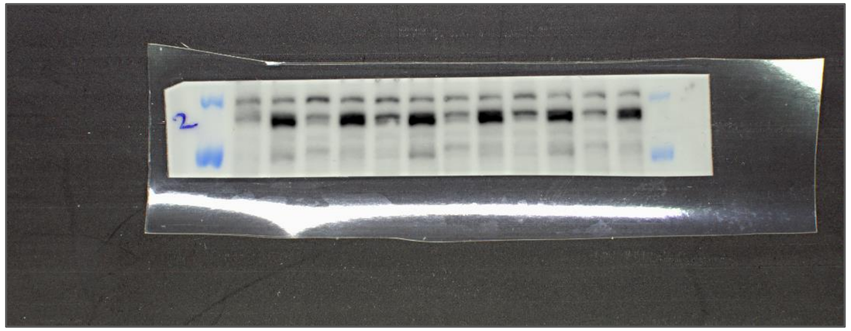

Tubulin

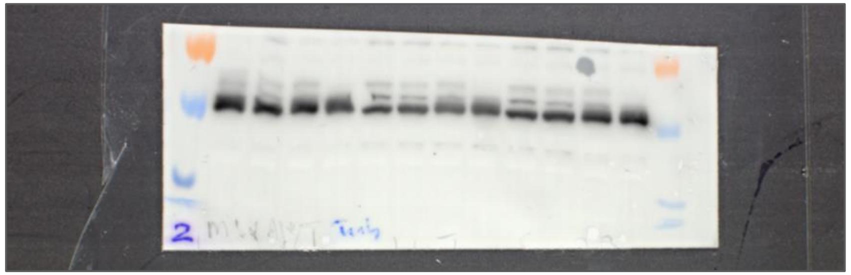

Full length Western Blots (Figure 3)

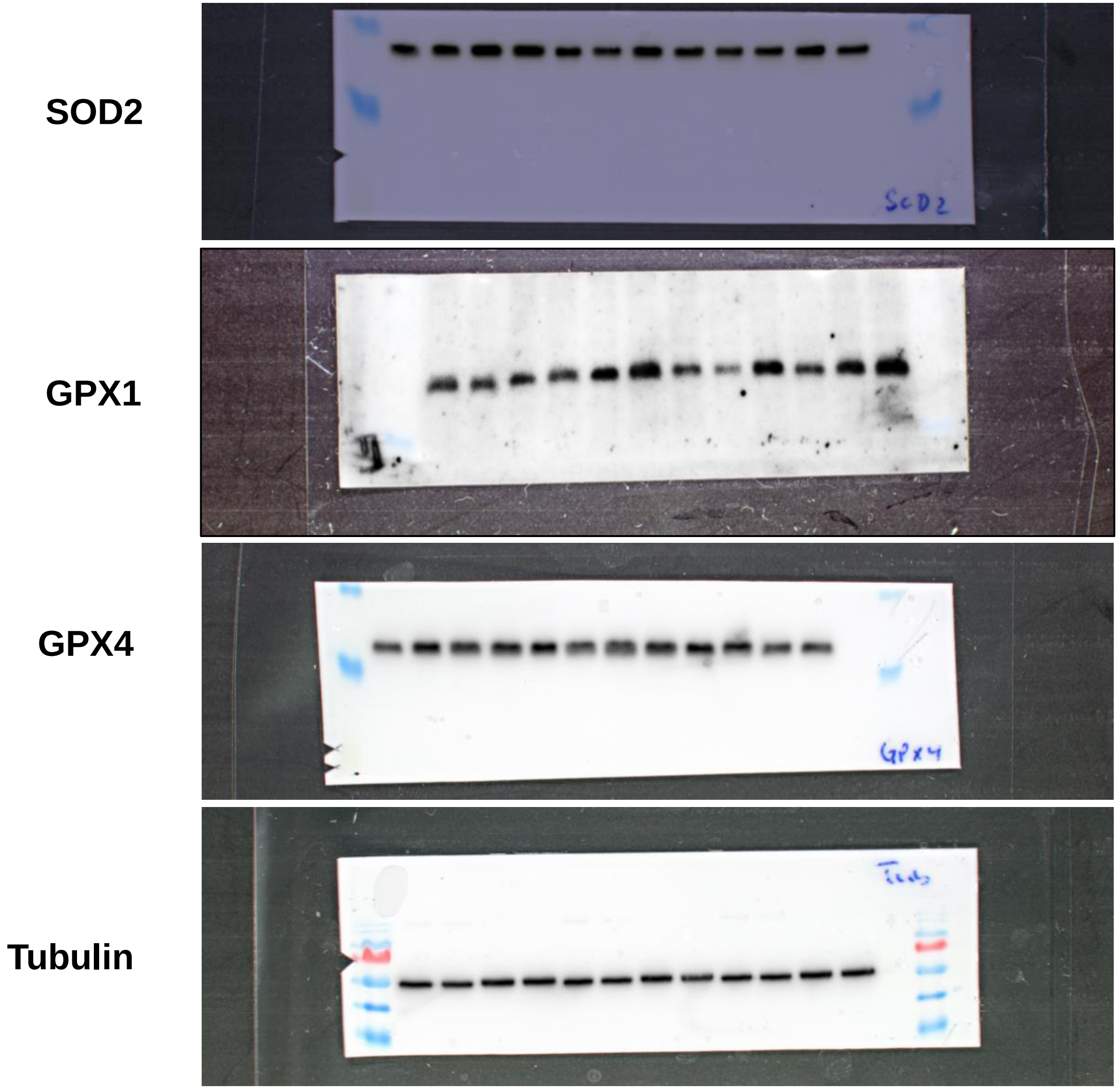

Full length Western Blots (Figure 4)

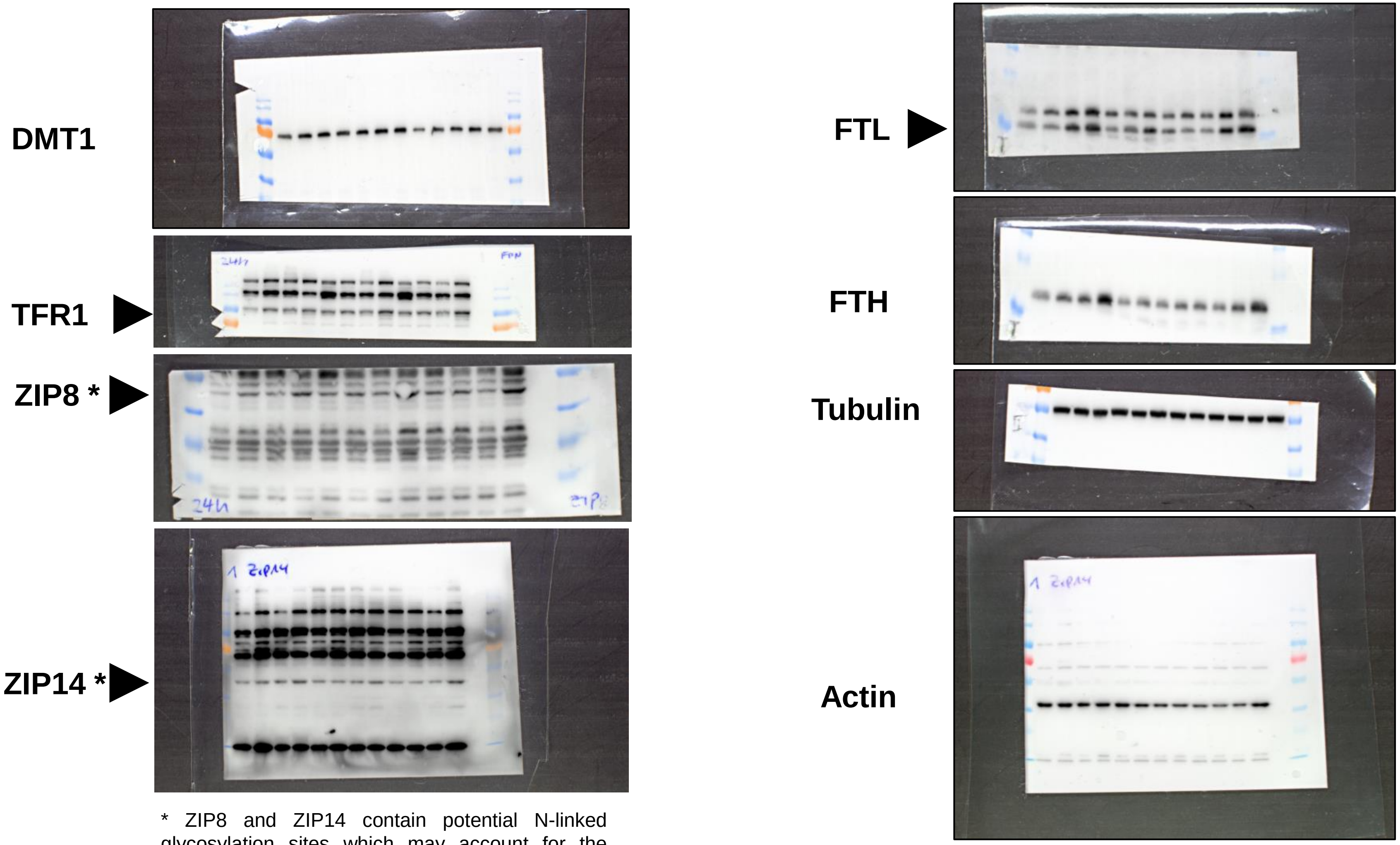

* ZIP8 and ZIP14 contain potential N-linked glycosylation sites which may account for the presence of multiple molecular weights. All analyses were conducted based on the MW of the native protein.
